# Anamorelin Efficacy in Non–Small‐Cell Lung Cancer Patients With Cachexia: Insights From ROMANA 1 and ROMANA 2

**DOI:** 10.1002/jcsm.13732

**Published:** 2025-02-24

**Authors:** Barry J. A. Laird, Richard Skipworth, Philip D. Bonomi, Marie Fallon, Stein Kaasa, Ruben Giorgino, Donald C. McMillan, David C. Currow

**Affiliations:** ^1^ Institute of Genetics and Cancer University of Edinburgh, Western General Hospital Edinburgh UK; ^2^ Clinical Surgery Royal Infirmary of Edinburgh University of Edinburgh Edinburgh UK; ^3^ Department of Internal Medicine Rush University Medical Center Chicago Illinois USA; ^4^ Department of Oncology Oslo University Hospital and University of Oslo Oslo Norway; ^5^ Formerly of Research and Development Innovation and Strategy Department Helsinn Healthcare SA Lugano Switzerland; ^6^ Academic Unit of Surgery, School of Medicine University of Glasgow Glasgow UK; ^7^ Faculty of Science, Medicine and Health University of Wollongong Wollongong Australia

**Keywords:** cachexia, cancer, modified Glasgow Prognostic Score, ROMANA 1, ROMANA 2, systemic inflammatory response, trials

## Abstract

**Background:**

Cancer cachexia presents a significant challenge, but the ghrelin agonist anamorelin shows promise as a potential treatment. This study examined whether the baseline systemic inflammatory response (SIR) (measured by the modified Glasgow Prognostic Score [mGPS]), low BMI or greater weight loss, was associated with a differential treatment effect of anamorelin in people with cachexia and non–small‐cell lung cancer (NSCLC).

**Methods:**

ROMANA 1 and ROMANA 2 were double‐blind, placebo‐controlled, randomised Phase 3 trials that enrolled people with inoperable stage III/IV NSCLC with cachexia (≥ 5% weight loss within 6 months or body mass index [BMI] < 20 kg/m^2^). Patients were randomised 2:1 to anamorelin 100 mg once daily or placebo, for 12 weeks. This is a post hoc analysis of efficacy endpoints (body weight and body composition: lean body mass [LBM] and fat mass [FM]), stratified by baseline mGPS, BMI and weight loss and measured in the modified intent‐to‐treat pooled population.

**Results:**

Seven hundred ninety‐five patients had available data. Anamorelin improved body weight (*p* < 0.001) and body composition parameters (LBM and FM, *p* < 0.01) in all mGPS groups. In patients with mGPS = 2, anamorelin increased weight > 5% and improved hand grip strength (HGS) and the Functional Assessment of Anorexia/Cachexia Therapy Anorexia/Cachexia Subscale (FAACT A/CS). In patients with BMI < 20 kg/m^2^ at baseline or weight loss ≥ 10% in the prior 6 months, anamorelin led to significant increases in body weight from baseline (*p* < 0.001) versus placebo. Patients with weight loss ≥ 10% in the prior 6 months showed the highest improvements in LBM (*p* < 0.001). Patients with BMI < 20 kg/m^2^ at baseline showed the highest improvements in FM (*p* < 0.001).

**Conclusion:**

Anamorelin improved body composition parameters in all patients, as well as physical function and symptom burden, particularly in patients with systemic inflammation, BMI < 20 kg/m^2^ and weight loss ≥ 10%. These results highlight that the anabolic mechanisms of anamorelin are more effective in high‐risk groups.

**Trial Registration:**

NCT identifiers: ROMANA 1: NCT01387269; ROMANA 2: NCT01387282.

## 1 Introduction

Cancer anorexia–cachexia syndrome (CACS) is a complex, systemic metabolic syndrome characterised by reduced appetite, weight loss (WL), decreased physical functioning and reduced response to systemic anticancer therapy [1, 2, 3]. Its prevalence is high in patients with advanced cancers, reaching up to 80% in patients with advanced non–small‐cell lung cancer (NSCLC) [4] and having negative effects on quality of life [5]. A body mass index (BMI) < 20 kg/m^2^ or an unintentional WL of ≥ 5% over 6 months are primary clinical manifestations of CACS in these patients [1].

Anamorelin is a highly selective ghrelin receptor agonist able to activate pathways controlling appetite, body weight and lean body mass (LBM) [6]. The results of two randomised controlled trials (ROMANA 1, *n* = 484; ROMANA 2, *n* = 495) examining the effect of anamorelin on cachexia in patients with NSCLC reported a significant increase in median LBM and body weight of patients following treatment. The observed changes in handgrip strength (HGS), a co‐primary endpoint, did not reach statistical significance in either trial [7]. Similar results were seen in a similar study in Japan (*n* = 174) [8, 9, 10]. The secondary endpoints of anorexia, concerns and body weight were positive in these trials, as were long‐term safety data [7, 8, 11, 12]. Despite these positive findings and approval in Japan, anamorelin has not received regulatory approval as a treatment for cancer cachexia elsewhere, with a principal reason being the failure to meet the co‐primary endpoints of improvement in HGS [13].

The pathophysiology of CACS is complex and the systemic inflammatory response (SIR) is central to the genesis and maintenance of cachexia [14]. Measures of the SIR, such as the modified Glasgow Prognostic Score (mGPS), have been recommended in the European Society for Clinical Nutrition and Metabolism (ESPEN) guidelines for cancer‐related malnutrition [15] and the European Society for Medical Oncology (ESMO) cachexia guidelines [16]. The SIR also influences the development of metastases [17], quality of life [18, 19] and has established prognostic value independent of tumour stage and performance status (PS) [20, 21]. Stratification by mGPS at baseline might prove highly useful in understanding outcomes in cancer cachexia clinical trials.

The efficacy of cancer therapies has been demonstrated to be influenced by the SIR, with calls for a priori stratification in clinical trials based on measures of the SIR [22], including anamorelin in order to identify any subgroups where anamorelin may be more effective [23]. It is also noted that anamorelin is likely to partially exhibit its effect through an anti‐inflammatory mechanism by attenuation of endotoxin‐induced anorexia [24], inhibition of nuclear factor‐κB as well as other inflammatory pathways [25, 26]. Consequently, there is a rationale for anamorelin treatment most likely to be efficacious in the presence of sustained systemic inflammation. There is also a sound rationale that the timing of cachexia interventions is important; for example, omega 3 fatty acids may be more efficacious earlier in the cachexia trajectory, while agents such as anamorelin may be more effective later on.

Additionally, it has been widely advocated in multiple learned guidelines that nutritional support should be given for patients with cancer cachexia. The rationale for this has primarily been based on first principles and consensus rather than evidence. It is plausible that cancer cachexia treatments would be more effective in individuals who have lost more weight, as they often have a greater degree of muscle wasting and nutritional depletion. Yet, three large systematic reviews have failed to demonstrate that increasing nutritional intake improves weight or survival; however, these did not specifically look at appetite stimulants and also predate the advent of trials of ghrelin agonists [27, 28, 29]. In the original ROMANA analyses, all patients had WL of > 5%; however, additional subgroup analyses in patients with either WL ≥ 10% or BMI ≤ 18.5 kg/m^2^ suggested that anamorelin was more effective than placebo in these patients. This would align with its anabolic mode of action but requires further examination.

Therefore, the aim of the present study was to examine whether the baseline SIR and the degree of WL/BMI were associated with differential treatment effects of anamorelin in people with NSCLC and cachexia enrolled in the ROMANA 1 and ROMANA 2 trials.

## 2 Methods

### 2.1 Design and Participants

The study design and participants of the ROMANA 1 and ROMANA 2 randomised controlled trials have been previously described [7]. In brief, these were two identical Phase 3 double‐blind, randomised trials of anamorelin versus placebo in patients with advanced NSCLC and cachexia. These trials took place across 93 hospital and community sites internationally and complied with International Ethical Guidelines for Biomedical Research Involving Human Subjects, Good Clinical Practice and the Declaration of Helsinki. All patients provided written informed consent, and full ethical approval was given by the institutional review boards at each participating centre. The present study is a post hoc pooled analysis of efficacy endpoints performed on the modified intent‐to‐treat (mITT—completed the trial with data available on reported data herein) population from ROMANA 1 and ROMANA 2. Ethical approval given for the initial trials (ROMANA 1 and ROMANA 2) included consent for data to be used in additional analyses. This subsequent analysis was conducted by Helsinn (Silvia Olivari Tilola), and approval was given for these data to be published.

Eligible patients met the following key criteria: adults (≥ 18 years of age), histologically confirmed unresectable stage III or IV NSCLC, had cachexia (defined as involuntary WL of ≥ 5% within the previous 6 months or BMI < 20 kg/m^2^) and Eastern Cooperative Oncology Group (ECOG) PS of 0–2. Patients were permitted to receive concomitant chemotherapy or radiotherapy. Following consent, patients were randomised (2:1) to anamorelin hydrochloride or placebo, using geographical region, cancer treatment status and WL as stratification factors.

### 2.2 Procedures

Participants took one capsule daily for 12 weeks. Dual‐energy X‐ray absorptiometry was used to measure body composition parameters (LBM, appendicular LBM [aLBM] and fat mass [FM]) at baseline and at 6 and 12 weeks. A calibrated scale was used to measure body weight, and hand‐held dynamometers were used for quantifying HGS. The Functional Assessment of Anorexia/Cachexia Therapy Anorexia/Cachexia Subscale (FAACT A/CS, score range: 0–48) and Functional Assessment of Chronic Illness Therapy—Fatigue (FACIT‐F, score range: 0–52) instruments were used to assess symptom burden, with higher scores representing a lower symptom burden. Body weight and symptom burden were both assessed at baseline and every 3 weeks.

### 2.3 Outcomes

The objectives of this study were to evaluate changes from baseline in body weight, body composition parameters, HGS and symptom burden at the end of study (EOS, Week 12), by mGPS, BMI and WL, at baseline.

### 2.4 Statistical Analysis

Data from the mITT population from both ROMANA 1 and ROMANA 2 were pooled and used for all efficacy analyses. Stratification was performed according to the mGPS, BMI and WL.

The mGPS was calculated using C‐reactive protein (CRP) and albumin levels, quantified in the venous blood taken by sampling at the point of consent. The limit of C‐RP detection was < 5 mg/L, and all samples were analysed for determination of C‐RP and albumin levels at a central laboratory. The mGPS was calculated as follows: Patients with C‐RP levels ≤ 10 mg/L were considered to have an mGPS of 0, patients with levels of circulating C‐RP > 10 mg/L had an mGPS of 1 and patients with C‐RP levels > 10 mg/L and albumin levels < 35 g/L had an mGPS of 2 [30]. Low BMI was categorised as < 20 kg/m^2^, and a high degree of WL was defined as ≥ 10% in the previous 6 months.

Descriptive statistics by mGPS, BMI and WL group were used to summarise demographics and baseline characteristics of patients. Means, standard deviations and two‐sided 95% confidence intervals (CIs) were calculated for comparisons between anamorelin and placebo. No hierarchy was applied to end‐points, and all statistical analyses were post hoc, with no formal statistical comparisons performed; all listed *p* values are nominal. The FAACT A/CS and FACIT‐F items were assessed at individual level and overall. Data analyses were performed with the SAS for Windows statistical package (Version 9.2 or above; SAS Institute Inc., Cary, NC).

## 3 Results

### 3.1 Patient Demographics and Baseline Characteristics

**FIGURE 1:**
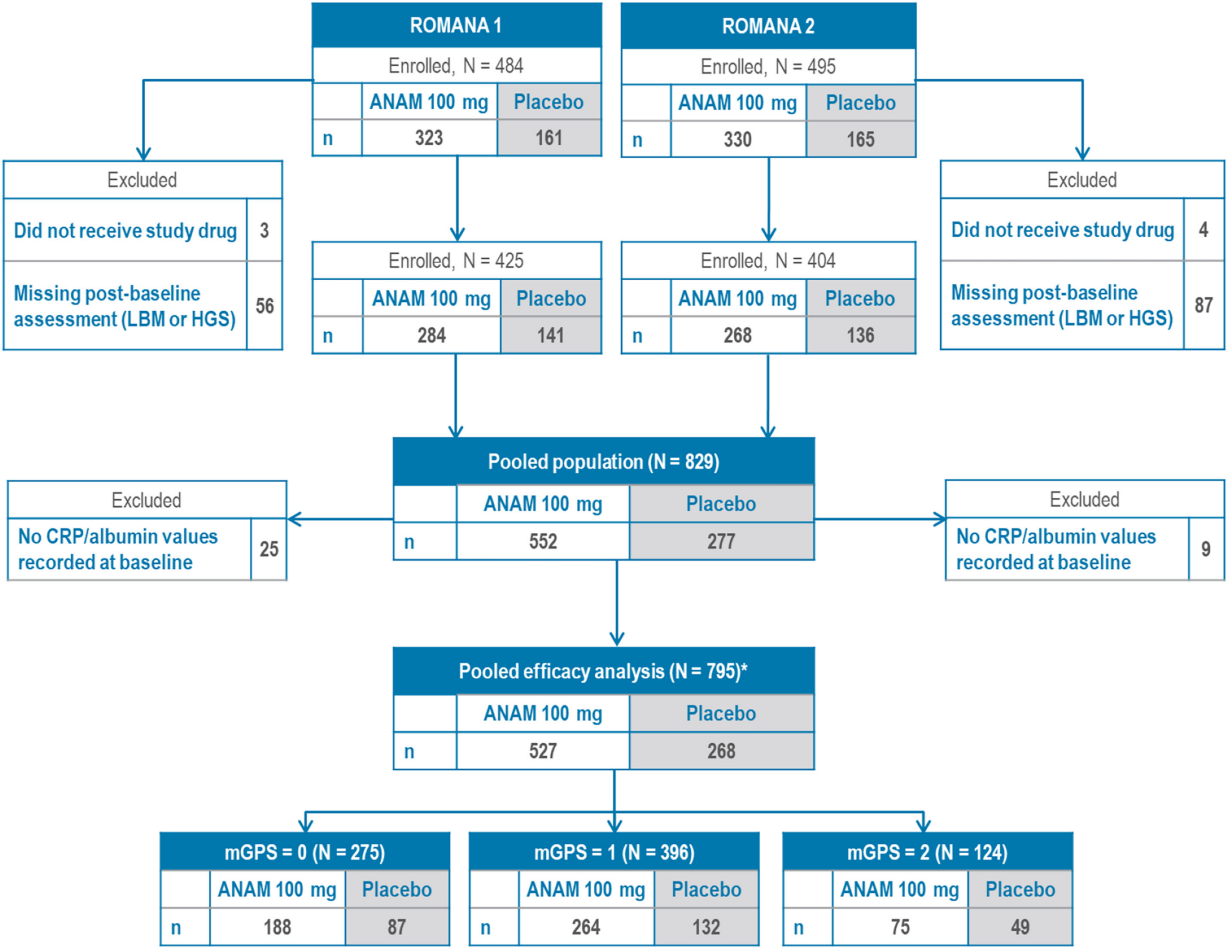
Pooled ROMANA 1 and ROMANA 2 efficacy analysis: Patient disposition per mGPS at baseline. *mITT population. ANAM: anamorelin; CRP: C‐reactive protein; HGS: handgrip strength; LBM: lean body mass; mGPS: modified Glasgow Prognostic Score; mITT: modified intent‐to‐treat.

Figure 1 shows the mITT population on which the post hoc pooled efficacy analysis was performed and consisted of 795 patients, where data on the mGPS were available. Of those, 275 patients had a mGPS of 0, 396 had a mGPS of 1 and 124 had a mGPS of 2 at baseline.

**FIGURE 2:**
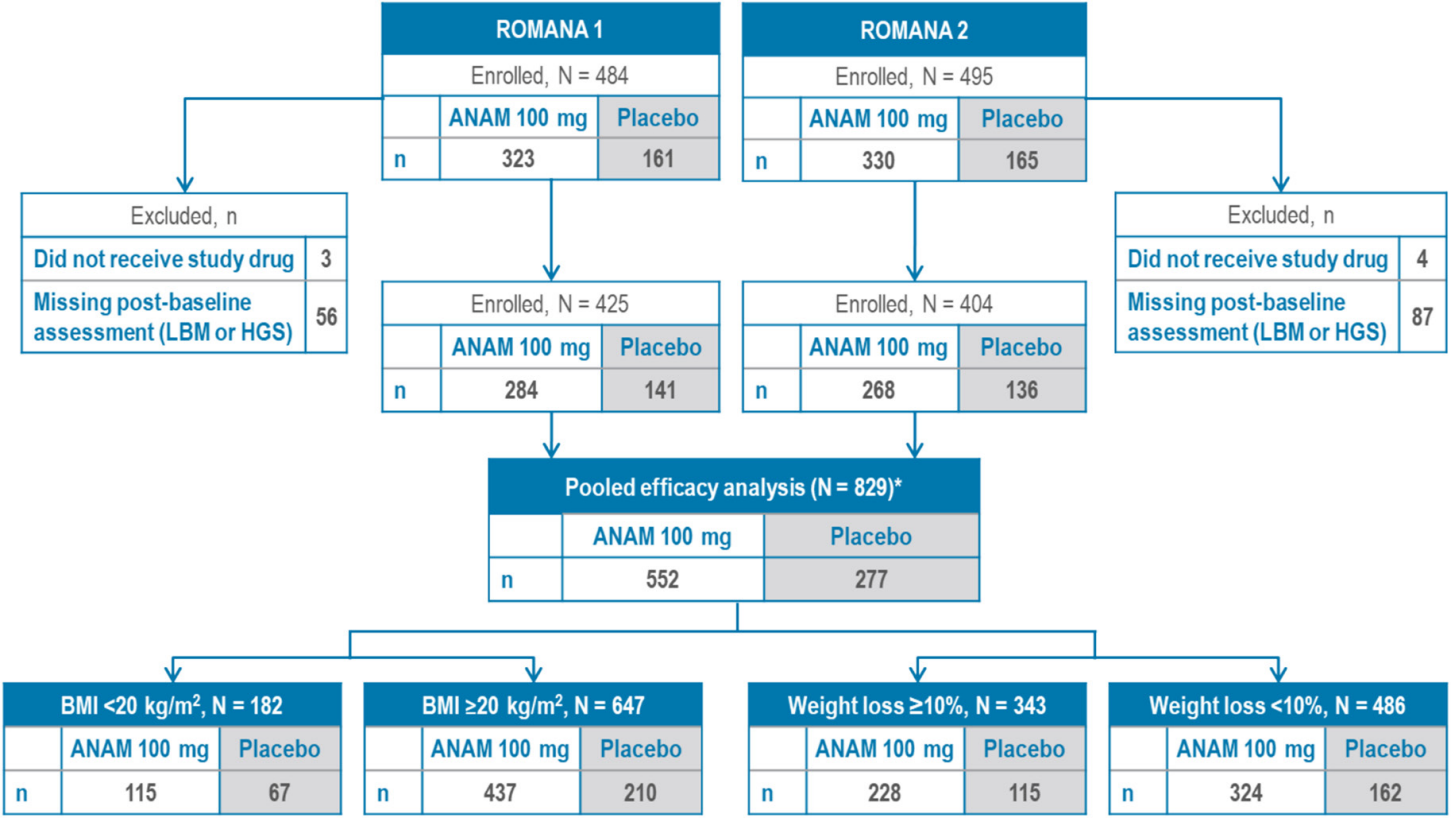
Pooled ROMANA 1 and ROMANA 2 efficacy analysis: Patient disposition per BMI and weight loss at baseline. *mITT population. ANAM: anamorelin; BMI: body mass index; HGS: handgrip strength; LBM: lean body mass; mITT: modified intent‐to‐treat.

Figure 2 shows the mITT population on which the post hoc pooled efficacy analysis was performed consisted of 829 patients, where data on BMI and WL were available. Of those, 182 patients had a BMI < 20 kg/m^2^, and 343 had WL ≥ 10%.

Table 1 shows patient demographics and baseline characteristics per mGPS, BMI < 20 kg/m^2^ and WL ≥ 10%. The mean age was 62.2 years (SD 8.9), and 209 (25.2%) were female. Differences according to mGPS status were observed in the following baseline characteristics: time from initial diagnosis (from 11.1 [mGPS = 2] to 22.4 months [mGPS = 0]), Karnofsky Performance Scale (KPS) score ≤ 70% (from 12% [mGPS = 0] to 31.5% [mGPS = 2] of patients). In summary, patients with an mGPS of 2 had lower PS and an overall shorter time from diagnosis than those with an mGPS of 0.

**TABLE 1:** Patient demographics and baseline clinicopathological characteristics according to mGPS, BMI and weight loss at baseline (mITT population).

	Overall (*N* = 829)	mGPS = 0 (*N* = 275)	mGPS = 1 (*N* = 396)	mGPS = 2 (*N* = 124)	BMI < 20 kg/m^2^ at baseline (*N* = 182)	Weight loss ≥ 10% in prior 6 months (*N* = 343)
Mean age, years (SD)	62.2 (8.9)	62.6 (8.8)	61.2 (8.4)	63.7 (9.3)	61.1 (9.1)	62.8 (9.1)
Female, *n* (%)	209 (25.2)	88 (32.0)	78 (19.7)	30 (24.2)	53 (29.1)	102 (29.7)
Ethnic origin, *n* (%)						
Asian	1 (0.1)	0	1 (0.2)	0	0	0
Black or African‐American	4 (0.5)	1 (0.3)	2 (0.5)	1 (0.8)	3 (1.6)	3 (0.9)
Native Hawaiian/Pacific Islander	1 (0.1)	1 (0.3)	0	0	1 (0.5)	1 (0.3)
Other	5 (0.6)	0	1 (0.2)	0	1 (0.5)	2 (0.6)
White	4 (0.5)	270 (98.2)	392 (99.0)	122 (98.4)	177 (97.3)	337 (98.3)
Baseline KPS ≤ 70, *n*/*N* valid (%)	204/824 (24.8)	33 (12.0)	81 (20.5)	39 (31.5)	70/179 (39.1)	110/341 (32.3)
Disease stage at study entry, *n* (%)						
IIIA	73/827 (8.8)	26 (9.5)	36 (9.1)	5 (4.0)	21/181 (11.6)	32/341 (9.4)
IIIB	157/827 (19.0)	52 (18.9)	80 (20.2)	19 (15.3)	32/181 (17.7)	64/341 (18.8)
IV	597/827 (72.2)	195 (70.9)	280 (70.7)	100 (80.6)	128/181 (70.7)	245/341 (71.8)
Mean time from initial tumour diagnosis, months (Q1–Q3)	18.2 (3.3–20.7)	22.4 (3.7–28.2)	17.4 (4.2–19.5)	11.1 (2.0–13.6)	14.6 (3.7–16.9)	15.3 (2.5–17.6)
Chemotherapy/radiotherapy to be started within 14 days of randomisation, *n* (%)	709 (85.5)	228 (82.9)	346 (87.4)	105 (84.7)	146 (80.2)	280 (81.6)
Concomitant use of opioids, *n* (%)	247 (29.8)	62 (22.6)	114 (28.8)	59 (47.6)	90 (49.5)	133 (38.8)
Weight loss ≤ 10%, *n* (%)	343 (41.4)	190 (69.1)	218 (55.1)	54 (43.5)	118 (64.8)	343 (100.0)
Weight loss > 10%, *n* (%)	486 (58.6)	85 (30.9)	178 (44.9)	70 (56.5)	64 (25.2)	0 (0.0)
Mean body weight, kg (SD)	66.44 (13.21)	66.9 (13.66)	67.4 (12.73)	63.0 (13.77)	51.91 (7.29)	61.71 (12.92)
Mean LBM, kg (SD)	45.31 (8.26)	44.9 (8.64)	46.2 (7.78)	44.5 (8.54)	39.94 (6.69)	42.95 (8.01)
Mean aLBM, kg (SD)	19.25 (4.26)	19.3 (4.59)	19.7 (3.95)	18.4 (4.25)	16.28 (3.37)	17.86 (4.03)
Mean FM, kg (SD)	18.78 (8.09)	19.4 (8.06)	19.0 (7.80)	16.3 (8.01)	9.93 (3.34)	16.42 (7.62)
Mean HGS non‐dominant arm, kg (SD)	31.43 (11.28)	32.2 (11.74)	32.7 (10.92)	27.2 (9.91)	28.19 (9.54)	28.33 (10.73)
Mean FAACT A/CS score (SD)	29.64 (8.42)	31.6 (7.92)	29.5 (8.16)	25.5 (8.77)	26.21 (8.43)	26.95 (8.82)
Mean FACIT‐F domain score (SD)	30.47 (10.48)	32.4 (9.74)	30.6 (10.21)	25.4 (10.95)	28.52 (11.49)	28.46 (11.06)

Abbreviations: aLBM: appendicular lean body mass; BMI: body mass index; FAACT A/CS: Functional Assessment of Anorexia/Cachexia Therapy Anorexia/Cachexia Subscale; FACIT‐F: Functional Assessment of Chronic Illness Therapy—Fatigue; FM: fat mass; HGS: handgrip strength; KPS: Karnofsky performance status; LBM: lean body mass; *N* valid: number of valid measurements; Q: quartile; SD: standard deviation.

For patients with a BMI < 20 kg/m^2^, the mean time from initial diagnosis was 14.6 months (compared to 18.2 months overall population), and 39.1% had a KPS of ≤ 70 (compared to 24.8% in the overall population).

For patients with WL ≥ 10%, the mean time from initial diagnosis was 15.3 months (compared to 18.2 months overall population), and 32.3% had a KPS of ≤ 70 (compared to 24.8% in the overall population).

### 3.2 Efficacy Analyses

**FIGURE 3:**
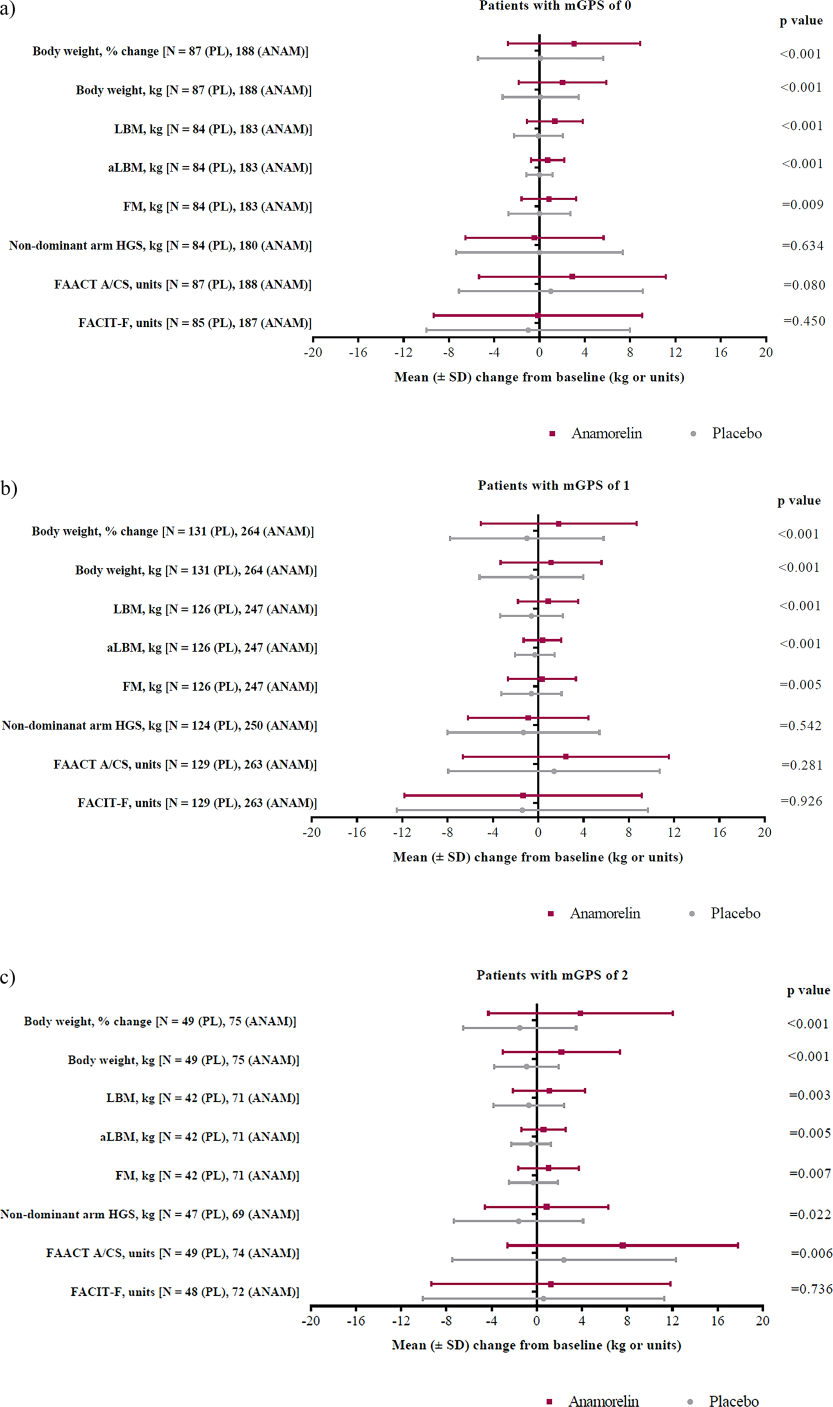
(a–c) Mean changes from baseline in body weight (percent change and absolute values), body composition parameters (LBM, aLBM and FM), non‐dominant arm HGS and patient‐reported outcomes (FAACT A/CS and FACIT‐F) at the end of study per treatment arm, in patients with (a) mGPS of 0, (b) mGPS of 1 and (c) mGPS of 2 (mITT population).

Figures 3a‐c shows the effects of anamorelin versus placebo on the mean changes from baseline in body weight, body composition and non‐dominant HGS per mGPS category. Treatment with anamorelin had a significant positive effect on body weight (absolute and percentage change, *p* < 0.001) and body composition parameters (LBM, aLBM and FM, *p* < 0.01), in all three mGPS groups. The magnitude of the anamorelin treatment effect (except for the FACIT‐F subscale) was most pronounced in patients with a mGPS of 2. In this patient group, the changes from baseline in the non‐dominant arm HGS and FAACT A/CS score were statistically significant following anamorelin treatment versus placebo (Figure 3c). In patients with a mGPS of 0 or 1, the changes in the non‐dominant arm HGS and FAACT A/CS score did not reach statistical significance (Figure 3a‐b). The percentage change in body weight compared to placebo was 3.00% (1.53–4.47, *p* < 0.001), 2.77% (1.33–4.21, *p* < 0.001) and 5.40% (2.82–7.97, *p* < 0.001) per mGPS 0, 1 and 2, respectively. The change in LBM compared to placebo was 1.43 (0.81–2.04, *p* < 0.001), 1.46 (0.88–2.04, *p* < 0.001) and 1.84 kg (0.62–3.06, *p* = 0.003) per mGPS 0, 1 and 2, respectively.

**FIGURE 4:**
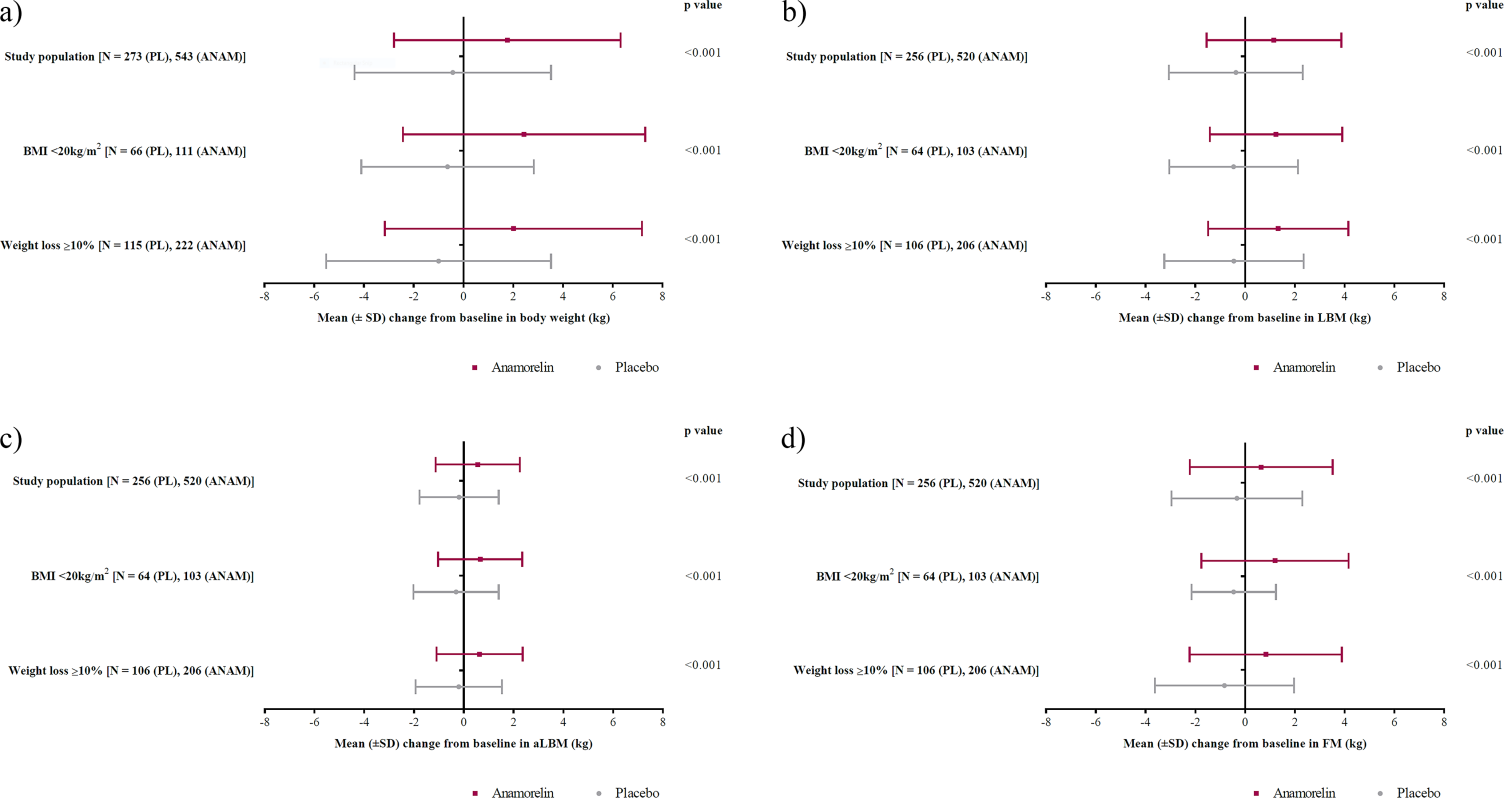
Mean changes from baseline in (a) body weight, (b) LBM, (c) aLBM and (d) FM at the end of study by patient subgroup and overall, in each treatment arm, in patients with BMI < 20 kg/m^2^ and weight loss ≥ 10% (mITT population).

Figure 4a–d shows treatment effects of anamorelin versus placebo on the mean changes from baseline in body weight, LBM, aLBM and FM based in those patients with a BMI < 20 kg/m^2^ and WL ≥ 10%. In the overall population, and in patients with BMI < 20 kg/m^2^ at baseline or WL ≥ 10% in the prior 6 months, anamorelin versus placebo led to significant increases in body weight from baseline (*p* < 0.001; Figure 4a). Treatment with anamorelin led to significant improvements in all body composition parameters (LBM, aLBM and FM) in both groups. Patients with WL ≥ 10% in the prior 6 months showed the highest improvements in LBM (*p* < 0.001; Figure 4b). Patients with BMI < 20 kg/m^2^ at baseline showed the highest improvements in aLBM (*p* < 0.001; Figure 4c) and FM (*p* < 0.001; Figure 4d). There were no statistically significant differences in HGS between anamorelin and placebo per BMI or WL category (data not shown).

In the overall population, and in patients with BMI < 20 kg/m^2^ at baseline or WL ≥ 10% in the prior 6 months, in comparison to placebo, anamorelin led to significant increases in body weight from baseline at EOS (2.19, 3.09 and 3.01, respectively; 95% CI: 1.56–2.83, 1.73–4.44 and 1.89–4.13, respectively; *p* < 0.001). Patients with BMI < 20 kg/m^2^ at baseline showed the highest improvements in aLBM (0.97; 95% CI: 0.43–1.50; *p* < 0.001) and FM (1.66; 95% CI: 0.86–2.46; *p* < 0.001). Patients with WL ≥ 10% in the prior 6 months showed the highest improvements in LBM (1.78; 95% CI: 1.12–2.44; *p* < 0.001).

We did not undertake adverse events analysis in the subgroups as the overall safety data from the original trials was reassuring. The most common treatment‐related events (> 10% incidence of Grade 1 or 2 or presence of any Grade 3 or 4 event) were diabetes and hyperglycaemia, which were equivocal between anamorelin and placebo arms. To illustrate this, in ROMANA 1, for hyperglycaemia, this was 1/320 (< 1%) in the anamorelin arm versus 1/161 (< 1%) in the placebo arm events. Remaining adverse events were less prevalent and similar in terms of frequency, between the anamorelin and placebo arms. In contrast, other trials investigating anamorelin have reported higher rates of adverse events compared to placebo. These include first‐degree atrioventricular block (6%), rash (6%), elevated γ‐glutamyltransferase levels (3.6%), hyperglycaemia (2.4%–4%), oedema (2.4%–4%) and hepatic dysfunction (6.4%) [9, 11]. While the overall adverse event profile remains encouraging, it is important to recognise that some patients may experience some adverse effects.

## 4 Discussion

The results of this post hoc analysis, based on combined data from the ROMANA 1 and ROMANA 2 trials, reveal compelling evidence for the efficacy of anamorelin in addressing body weight and composition in patients with NSCLC who have cachexia. Specifically, the study highlights that anamorelin exhibits the most pronounced improvements in these parameters, compared to placebo, particularly in individuals with systemic inflammation (as indicated by mGPS 1 and 2), a BMI < 20 kg/m^2^ or WL ≥ 10%. Notably, the presence of a SIR at the outset of treatment serves as a predictive marker for the differential response to anamorelin therapy in people with NSCLC and cachexia. Among those with a mGPS of 2, improvements in function (HGS—recognised as an optimal measure of physical function [31]) and symptoms are observed alongside improvements in body weight and composition metrics. Of particular note was that in mGPS 2, percentage weight change improved by > 5%, which is clinically significant. Moreover, even in patients grappling with severe manifestations of cancer cachexia—exemplified by a low BMI and substantial WL—anamorelin demonstrates efficacy in promoting favourable alterations in body composition. These observations are aligned with recent work by Takayama et al. where post hoc analyses (*n* = 193) revealed that anamorelin was potentially more effective in similar subgroups [32].

The methodology employed for the analyses, utilising WL, BMI and mGPS stratification, although conducted post hoc, corresponds with the GLIM diagnosis of cachexia, which involves considering both phenotypic and etiologic criteria [33]. One could postulate that combining WL with high or low mGPS, or BMI with mGPS, might effectively identify individuals with established cachexia who are most likely to derive benefit from intervention.

These findings underscore the dual anti‐inflammatory and anabolic mechanisms of anamorelin and suggest patient subgroups most likely to benefit from its therapeutic effects. Although the role of ghrelin in hunger stimulation is well established, a link to the anti‐inflammatory mechanism is not fully established in the clinical setting [34]. These observations build on preclinical data and highlight its potential therapeutic use in cancer cachexia where excessive inflammation is present. Cancer‐associated symptoms such as anorexia, WL and a decrease in physical function form a cluster, whereas fatigue, pain and depression form another cluster; however, all these manifestations are associated with the presence of a SIR [35, 36]. Evidence has accumulated that the SIR and malnutrition play a prognostic role in patients with various advanced cancers [14]. It has been extensively shown that the mGPS is prognostic in patients with NSCLC [37, 38], and in these patients, who present with more severe inflammation, treatment with combined anticancer therapies was shown to lead to a significantly greater overall survival than in patients with a lower mGPS [39]. Additionally, the mGPS has demonstrated predictive value concerning treatment outcomes, such as chemotherapy and immunotherapy in NSCLC. Ghrelin's neuroendocrine function suggests a potential anti‐inflammatory effect in systemically inflamed patients, which could explain its efficacy [40]. This association between metabolism and inflammation aligns logically and implies a neuroendocrine involvement in the pathogenesis of cancer cachexia among NSCLC patients. This concept underscores the significance of further exploration of hypothalamic inflammation in cachexia.

These results suggest that interventional therapies for cancer, and possibly for associated health issues such as cancer anorexia–cachexia, might be more efficacious in patients with a higher underlying inflammatory response, burdened by compounding disease and who generally present with a poorer overall outcome. In this study, anamorelin significantly improved the non‐dominant arm HGS and the FAACT A/CS score in patients with an mGPS of 2, in addition to improving body weight and body composition parameters in all three mGPS groups. While a similar trend for enhancement of non‐dominant arm HGS and FAACT A/CS score was also observed in patients with a mGPS of 0–1, presenting with a less prominent SIR, this did not reach statistical significance. Furthermore, the extent of weight improvement in patients with an mGPS of 0–1 was lower than in patients with an mGPS of 2, thus suggesting that treatment with anamorelin has the potential to reverse or attenuate pathologic WL. These findings suggest that patients exhibiting systemic inflammation may derive the greatest benefit from anamorelin, aligning with its known yet less recognised anti‐inflammatory mechanism. However, it is important to highlight that the potential mechanism of supplementary Ghrelin may not necessarily entail an anti‐inflammatory action, but rather, it could operate by outcompeting inflammatory molecules such as IL‐6 and LEAP‐2 at appetite centres in the brain [41]. Currently, we lack evidence regarding whether CRP levels decrease with Ghrelin treatment, which is crucial to confirming any potential anti‐inflammatory effects. Further research is warranted to determine the possibility that in patients experiencing a SIR, combining anamorelin with an anti‐inflammatory agent could enhance its effectiveness, akin to the practice of using low‐dose steroids alongside immunotherapy.

The anabolic mechanism of ghrelin is however well recognised and a large proportion of patients included in this pooled analysis had metastatic disease (72%) or severe anorexia–cachexia self‐reported concerns (FAACT A/CS score ≤ 30, 49%). Additionally, at baseline, 65% had evidence of a SIR (mGPS > 0), 22% were underweight (BMI < 20 kg/m^2^) and 41% had WL (≥ 10%) in the 6 months prior to enrolment. Despite the often‐severe nature of cancer cachexia in these patients, anamorelin demonstrated efficacy at improving body composition in all subgroups. It is unlikely that the changes in LBM were caused by confounding factors such as fluid retention; rather, the orexigenic and anabolic effects of anamorelin are consistent with its mechanism of action as a ghrelin receptor agonist [42]. Furthermore, the significant increase in FM provided objective evidence of improved appetite and energy consumption to complement the subjective evidence of an improved FAACT A/CS score.

Previously, an apparent disconnect between appetite and QoL improvements has been observed following treatment of people with advanced cancer with the appetite stimulant megestrol acetate [43]. Improvements in fatigue have also been difficult to demonstrate even in a patient population with less WL than that of the ROMANA 1 and ROMANA 2 trials [44]. In the present analysis, patients with BMI < 20 kg/m^2^ or with WL ≥ 10% in the prior 6 months experienced a significant increase in both LBM and FM concordant with a significant stimulation of appetite and a limited impact on fatigue overall. Particularly, the effect of anamorelin on anorexia–cachexia symptom burden and fatigue experienced by patients with BMI < 20 kg/m^2^ at baseline was much greater than in the overall population, with a statistically significant difference compared with placebo, which translated into a clinically meaningful improvement. Overall, these results suggest that patients with more advanced cachexia may still benefit from anamorelin treatment.

This study has limitations. Firstly, the efficacy analysis stratified per mGPS, BMI and WL group was conducted post hoc rather than being predefined, which restricts the interpretability of the findings. Additionally, the assessment of caloric intake and food diaries among patients was not incorporated into the study design. Examining potential changes in these parameters by mGPS group could have provided valuable supplementary insights. Therefore, it is advisable to include such assessments in the design of future randomised trials investigating drugs for the treatment of cancer anorexia–cachexia. Study participants were also eligible to receive various standard chemotherapy/radiotherapy regimens, potentially influencing the effects of anamorelin on the measured outcomes. Finally, no measurements were conducted to validate increased food intake with anamorelin, limiting the ability to draw conclusions regarding an objective treatment effect.

## 5 Conclusion

The findings of this post hoc analysis, drawing from the combined data of the ROMANA 1 and ROMANA 2 trials, provide evidence supporting the efficacy of anamorelin in addressing body weight and composition in patients with NSCLC and cachexia. The study underscores that anamorelin yields the most substantial enhancements in these parameters compared to placebo, especially in patients with systemic inflammation, a low BMI or significant WL. Notably, the presence of a SIR serves as a predictive indicator for the differential response to anamorelin therapy in cachectic NSCLC patients. Even in patients experiencing severe manifestations of cancer cachexia, anamorelin demonstrates efficacy in promoting favourable alterations in body composition.

## Conflicts of Interest

D.C.C. and D.C.M. have undertaken consultancy for Helsinn. R.S. has received personal fees for consultancy from Artelo, Actimed, Faraday and Helsinn. B.J.A.L. has received personal fees for consultancy from Artelo, Actimed, Faraday, Kyona Kirin, Helsinn and Toray.
